# Prognostic value of soluble endoglin in patients with septic shock and severe COVID-19

**DOI:** 10.3389/fmed.2022.972040

**Published:** 2022-08-31

**Authors:** Veronika Tomášková, Alexandra Mýtniková, Marcela Hortová Kohoutková, Ondřej Mrkva, Monika Skotáková, Michal Šitina, Kateřina Helánová, Jan Frič, Jiří Pařenica, Vladimír Šrámek, Martin Helán

**Affiliations:** ^1^Department of Anesthesiology and Intensive Care, St. Anne's University Hospital, Brno, Czechia; ^2^Faculty of Medicine, Masaryk University, Brno, Czechia; ^3^International Clinical Research Center, St. Anne's University Hospital, Brno, Czechia; ^4^Department of Cardiology, University Hospital Brno, Brno, Czechia; ^5^Department of Modern Immunotherapy Research, Institute of Hematology and Blood Transfusion, Prague, Czechia

**Keywords:** endoglin, COVID-19, sepsis, shock, endothelial dysfunction, biomarker, mortality

## Abstract

Sepsis is a clinical syndrome characterized by a dysregulated response to infection. It represents a leading cause of mortality in ICU patients worldwide. Although sepsis is in the point of interest of research for several decades, its clinical management and patient survival are improving slowly. Monitoring of the biomarkers and their combinations could help in early diagnosis, estimation of prognosis and patient's stratification and response to the treatment. Circulating soluble endoglin (sEng) is the cleaved extracellular part of transmembrane glycoprotein endoglin. As a biomarker, sEng has been tested in several pathologic conditions where its elevation was associated with endothelial dysfunction. In this study we have tested the ability of sEng to predict mortality and its correlation with other clinical characteristics in the cohort of septic shock patients (*n* = 37) and patients with severe COVID-19 (*n* = 40). In patients with COVID-19 sEng did not predict mortality or correlate with markers of organ dysfunction. In contrast, in septic shock the level of sEng was significantly higher in patients with early mortality (*p* = 0.019; AUC = 0.801). Moreover, sEng levels correlated with signs of circulatory failure (required dose of noradrenalin and lactate levels; *p* = 0.002 and 0.016, respectively). The predominant clinical problem in patients with COVID-19 was ARDS, and although they often showed signs of other organ dysfunction, circulatory failure was exceptional. This potentially explains the difference between sEng levels in COVID-19 and septic shock. In conclusion, we have confirmed that sEng may reflect the extent of the circulatory failure in septic shock patients and thus could be potentially used for the early identification of patients with the highest degree of endothelial dysfunction who would benefit from endothelium-targeted individualized therapy.

## Introduction

Under the current definition, sepsis is described as a life-threatening organ dysfunction that is caused by a deregulated immune response to infection (1). Sepsis may further develop into septic shock, a condition characterized by elevated blood lactate levels and persistent hypotension with adequate volume resuscitation. Sepsis/septic shock is increasing in incidence and has a continued high mortality rate (up to 40%); it represents the leading cause of death in ICU (intensive care unit) patients worldwide (2). The pathophysiological mechanisms leading to the development and progression of sepsis are very complex and have not yet been sufficiently clarified. The key role is played by the uncontrolled immunological response to the infection with the activation of pro-inflammatory and anti-inflammatory processes, which subsequently lead to the development of endothelial dysfunction, circulatory failure and results in organ dysfunction. However, many other mechanisms are involved in organ damage such as coagulopathy, oxidative stress, mitochondrial dysfunction, apoptosis, etc. (3, 4). The considerable number of factors involved, the range of different infectious agents and the different genotypes and phenotypes of patients determine the marked heterogeneity of sepsis. This heterogeneity accounts for the failures currently experienced in large randomized clinical trials testing new treatment options. Therefore, there is a great need to find appropriate biomarkers (and their combinations) to help stratify patients according to their prognosis and phenotypes into smaller groups that respond better to the tested medication (5).

COVID-19, caused by the SARS-CoV-2 virus, progresses in some patients to very severe respiratory failure due to ARDS (an incidence rate of ~5%) (6, 7). Although COVID-19 primarily damages lung tissue, it often manifests itself as a generalized disease showing endothelial damage, activation of inflammation and cytokine release, the development of disseminated intravascular coagulation, etc. (8). The severe course of COVID-19 falls within the definition of sepsis (9), and although it is a clinically distinct syndrome, many of the manifestations are the same as in sepsis: dysregulated immune response, cytokine storm, acute respiratory distress syndrome (ARDS), multiple organ dysfunction syndrome (MODS), hypercoagulable state, etc. (10, 11).

Endoglin (Eng) is an adhesion molecule, a sub-unit of the receptor system for TGF-β. It is the most frequently localized on the surface of endothelial cells, but its expression has been demonstrated in many other cell types, such as the cells of innate and adaptive immunity (12, 13). Therefore, Eng might be a factor which helps to shape the immune response on multiple levels (14). In the early phase of inflammation, it plays a regulatory role in the transendothelial migration of leukocytes by binding to leukocyte integrins (15). Its function is also associated with the differentiation process of hematopoietic cells (14, 16). Eng is also one of the factors in angiogenesis (including hypoxic neo-angiogenesis), as evidenced by the well-described phenotype of patients with hereditary hemorrhagic telangiectasia (type 1 HHT), which is caused by a mutation in the gene for Eng. This disease is primarily characterized by damage to the endothelium. However, infectious complications are the most common cause of death in these patients (35%) (17). Under certain conditions (ischemia, oxidative stress), the extracellular part of Eng is cleaved by metalloproteinase 14 to form circulating soluble endoglin (sEng) (18, 19). sEng is speculated to have rather antiangiogenic effects (20, 21) (unlike Eng). In the endothelium, it activates a pro-inflammatory phenotype (22) and has been considered an indicator of endothelial dysfunction in some conditions (23, 24).

Recently, we proved that sEng can be potentially used as a prognostic biomarker in patients suffering from septic shock (25). In COVID-19 patients, sEng levels have only been tested a few times with inconclusive results. Vieceli Dalla Sega et al. reported that sEng is increased in non-surviving COVID-19 patients at admission (26) whereas others did not show any difference between survivors and those who died (26). We assume that Eng may play a role in endothelial dysfunction as well as in the modulation of the immune response in septic shock and in the severe course of COVID-19.

In this trial, we sought to test the prognostic potential (in prediction of early (D3), D28 and/or D90 mortality) of sEng in a larger and more precisely defined cohort of patients suffering from septic shock and the cohort of patients with a severe COVID-19 and compare the result between these two diagnoses. Secondly, we wanted to determine whether sEng levels are correlated with other laboratory and clinical characteristics of patients including the source of sepsis, and thus bring new insights into sEng pathophysiology and function in critical infectious states.

## Methodology

### Patient cohorts

The trial included patients admitted to the intensive care unit (ICU) of the Department of Anesthesiology and Intensive Care at St. Anne's University Hospital in Brno with sepsis requiring administration of catecholamines (the septic shock cohort) and with a severe course of COVID-19 (the COVID-19 cohort). The trial was approved by the local Ethics Committee and patients were enrolled on the basis of informed consent. A total of 37 consecutive septic shock patients hospitalized in year 2019 were included in the septic shock cohort. All patients were treated according to the current recommendations for sepsis treatment (27). Inclusion criteria were: the need of artificial ventilation, the need of continuous catecholamine infusion to maintain blood pressure, early sepsis onset (antibiotic therapy not longer than 48 h before ICU admission), sepsis as a primary reason for ICU admission.

A total of 40 patients were included in the COVID-19 cohort, who were admitted to the ICU with a severe course of COVID-19 during the 2021 global pandemic. All enrolled patients required artificial pulmonary ventilation at the time of admission. Exclusion criteria were the same for both cohorts: ongoing cancer, chronic immunosuppressive therapy, age under 18 years, and current pregnancy.

### Sample processing

Venous blood samples were collected from patients during the first 24 h after ICU admission. Immediately after collection, the samples were transported to the laboratory and centrifuged. The plasma obtained in this manner was frozen and stored at −80°C until it was analyzed. The plasma sEng concentration was measured in triplicate using the Human Endoglin/CD105 Quantikine ELISA Kit (Sigma-Aldrich Ltd., Saint-Louis, Missouri, USA). The absorbance of the samples was measured using a Multiscan GO Microplate Spectrophotometer (Thermo Scientific) at 450 nm (wavelength correction of 540/570 nm) and the average value for three samples was calculated. Levels of routinely used biomarkers (C-reactive protein (CRP), creatinine, lactate, bilirubin) were analyzed by standard techniques in a hospital laboratory as part of routine daily patient diagnostics.

### Statistical analysis

A statistical analysis was performed in the R programming language [version R-4.0.5; R Core Team (2021). R Foundation for Statistical Computing, Vienna, Austria] in the R studio environment (version 1.4.1106). The data are presented as the median (1st quartile−3rd quartile). The statistical significance was set at *p* < 0.05. Spearman's rank correlation coefficient was used to measure the strength of the relationship between the two variables [levels of sEng were correlated to: age, Sequential organ failure assessment score (SOFA)], noradrenalin dose, leukocytes, lactate, CRP, creatinine, bilirubin and oxygenation index. The Mann–Whitney *U*-test was used to test the difference in continuous variables between the two groups; the Kruskal-Wallis test was used for 3 or more groups; and Fisher's exact test was used to test for statistical dependence.

## Results

A total of 37 patients were enrolled in the septic shock cohort and 40 in the COVID-19 cohort. Patient characteristics are described in Tables 1, 2. Briefly, the septic shock cohort was predominantly male (56.8%) with a median age of 73.0 (68.0–77.0), and the D28 mortality rate was 48.6%.

**Table 1:** Demographic and clinical characteristics of patients in septic shock cohort.

**Septic shock cohort**		**Total**	**Survivors**	**Non-survivors**	***p*-value**
*N*		37 (100%)	19 (51.4%)	18 (48.6%)	
Sex	Female	16 (43.2%)	8 (50.0%)	8 (50.0%)	1.000
	Male	21 (56.8%)	11 (52.4%)	10 (47.6%)	1.000
Age		73.0 (68.0–77.0)	68.0 (66.0–75.5)	74.5 (72.3–79.5)	**0.019**
BMI		27.7 (24.3–30.4)	28.9 (23.9–30.3)	27.4 (25.4–30.9)	0.867
Comorbidities	Total amount	2 (1–4)	2 (1–4)	2 (1–3)	0.486
IHD		12 (32.4%)	7 (58.3%)	5 (41.7%)	0.728
DM		15 (40.5%)	10 (66.7%)	5 (33.3%)	0.184
Asthma/COPD		8 (21.6%)	2 (25%)	6 (75%)	0.125
Peripheral ischemic disease	8 (21.6%)	7 (87.5%)	1 (12.5%)	**0.004**
Atherosclerosis-related diagnosis	23 (62.2%)	14 (60.9%)	9 (39.1%)	0.184
**Status at hospital admission**				
SOFA		11 (10–14)	12 (10–14.5)	11 (10–12.8)	0.669
CRP	[mg/L]	237.5 (109.7–361.6)	157.8 (112.5–366.7)	270.2 (105.7–334.8)	0.963
Leucocytes	[x10^9^/L]	14.6 (10.8–21.2)	13.3 (6.9–17.8)	18.7 (12.3–23.7)	**0.050**
Lactate	[mmol/L]	2.0 (1.4–3.0)	1.6 (1.2–2.1)	2.7 (2.0–4.8)	**0.003**
Noradrenalin dose	[ug/kg/min]	0.11 (0.06–0.21)	0.10 (0.03–0.23)	0.18 (0.08–0.21)	0.796
Oxygenation index	(PaO_2_/FiO_2_)	143.5 (122.8–214.4)	159.8 (140.4–205.8)	132.4 (118.6–219.5)	0.501
Creatinine	[μmol/L]	169.0 (113.0–258.0)	187.0 (110.5–480.5)	144.5 (113.3–235.0)	0.230
**Source of sepsis**					
	Pneumonia	15 (40.5%)	8 (53.3%)	7 (46.7%)	1.000
	Intra-abdominal	7 (18.9%)	4 (57.1%)	3 (42.9%)	1.000
	Urosepsis	6 (16.2%)	3 (50.0%)	3 (50.0%)	1.000
	Soft tissue infection	4 (10.8%)	1 (25.0%)	3 (75.0%)	0.340
	Mediastinitis	3 (8.1%)	2 (66.7%)	1 (33.3%)	1.000
	Other/Unknown	2 (5.4%)	1 (50.0%)	1 (50.0%)	1.000

Median (1st quartile−3rd quartile) values are presented for continuous variables, absolute and relative frequencies for binary variables. For the comparison of surviving and non-surviving patients, p-values of Mann–Whitney test are presented for continuous variables and p-values of Fisher's exact test are presented for binary variables.

BMI, body mass index; IHD, ischemic heart disease; DM, diabetes mellitus; COPD, chronic obstructive pulmonary disease; SOFA, sequential organ failure assessment; CRP, c-reactive protein. The bold values indicate statistical significance at *p* < 0.05.

**Table 2:** Demographic and clinical characteristics of patients in COVID-19 cohort.

**COVID-19 cohort**		**Total**	**Survivors**	**Non-survivors**	***p*-value**
*N*		40 (100%)	24 (60.0%)	16 (40.0%)	
Sex	Female	11 (27.5%)	7 (63.6%)	4 (36.4%)	1.000
	Male	29 (72.5%)	17 (58.6%)	12 (41.4%)	1.000
Age		63.5 (53.8–70.0)	62.5 (49.0–70.8)	64.0 (54.8–69.3)	0.793
BMI		28.1 (26.0–32.0)	28.1 (26.2–33.1)	28.6 (25.9–31.3)	0.581
Comorbidities	Total amount	1.0 (0–3)	1 (0–3)	1.5 (1–2.3)	0.670
IHD		5 (12.5%)	4 (80.0%)	1 (20.0%)	0.373
DM		9 (22.5%)	4 (44.4%)	5 (55.6%)	0.456
Asthma/COPD		6 (15.0%)	4 (66.7%)	2 (33.3%)	1.000
Atherosclerosis-related diagnosis	10 (25.0%)	5 (50.0%)	5 (50.0%)	0.717
**Status at hospital admission**				
SOFA		9 (7–11)	7.5 (7–10)	9 (7–12)	0.393
CRP	[mg/mL]	151.1 (67.6–248.7)	123.9 (47.5–210.7)	176.9 (121.5–251.6)	0.062
Leukocytes	[x10^9^/L]	10.7 (8.1–14.1)	10.7 (8.3–13.8)	10.8 (7.8–14.8)	0.934
Lactate	[mmol/L]	1.30 (1.10–1.90)	1.25 (1.10–1.93)	1.35 (1.08–1.58)	0.698
Noradrenaline dose	[ug/kg/min]	0.00 (0.00–0.09)	0.00 (0.00–0.05)	0.02 (0.00–0.15)	0.277
Oxygenation index	[PaO_2_/FiO_2_]	87.5 (63.5–116.3)	85.3 (65.4–101,7)	101.9 (53.9–130.7)	0.544
LISS		14.0 (12.8–15.0)	14.0 (13.0–14.3)	14.5 (12.0–15.0)	0.609
Creatinine	[μmol/L]	82.0 (73.8–105.8)	79.5 (73.0–96.3)	95.0 (78.8–124.5)	0.176
Bilirubin	[μmol/L]	8.6 (6.5–14.5)	7.6 (5.9–10.4)	10.7 (7.6–18.6)	0.075

Median (1st quartile−3rd quartile) values are presented for continuous variables, absolute and relative frequencies for binary variables. For the comparison of surviving and non-surviving patients, p-values of Mann–Whitney test are presented for continuous variables and p-values of Fisher's exact test are presented for binary variables.

BMI, body mass index; IHD, ischemic heart disease; DM, diabetes mellitus; COPD, chronic obstructive pulmonary disease; SOFA, sequential organ failure assessment; LISS, lung injury severity score; CRP, c-reactive protein.

The deceased patients were significantly older p74.5 (72.3–79.5) vs. 68.0 (66.0–75.5)] and had more pronounced leukocytosis [18.7 (12.3–23.7) vs. 13.3 (6.9–17.8) × 10^9^/L] and elevated lactate levels [2.7 (2.0–4.8) vs. 1.6 (1.2–2.1) mmol/L] at admission. There were no other statistically significant differences between the groups. All patients were mechanically ventilated upon admission and the SOFA score at admission was 11 (10–14). The most common comorbidities were obesity, diabetes mellitus, and ischemic heart disease and peripheral ischemic disease. The most common source of sepsis was pneumonia (40.5%; *n* = 15).

Similarly, in the COVID-19 cohort, males were overrepresented (72.5%), the median age was 63.5 years (53.8–70.0), and D28 mortality was 40.0%. There were no statistically significant differences between the patients who survived and those who died. Again, all patients were mechanically ventilated when admitted and the SOFA score at admission was 9 (7–11). The most common comorbidities were diabetes mellitus, asthma, and ischemic heart disease. Compared with the septic shock cohort, the patients were younger and had slightly fewer comorbidities [1.0 (0–3) vs. 2.0 (1–4); a non-significant difference].

**Figure 1:**
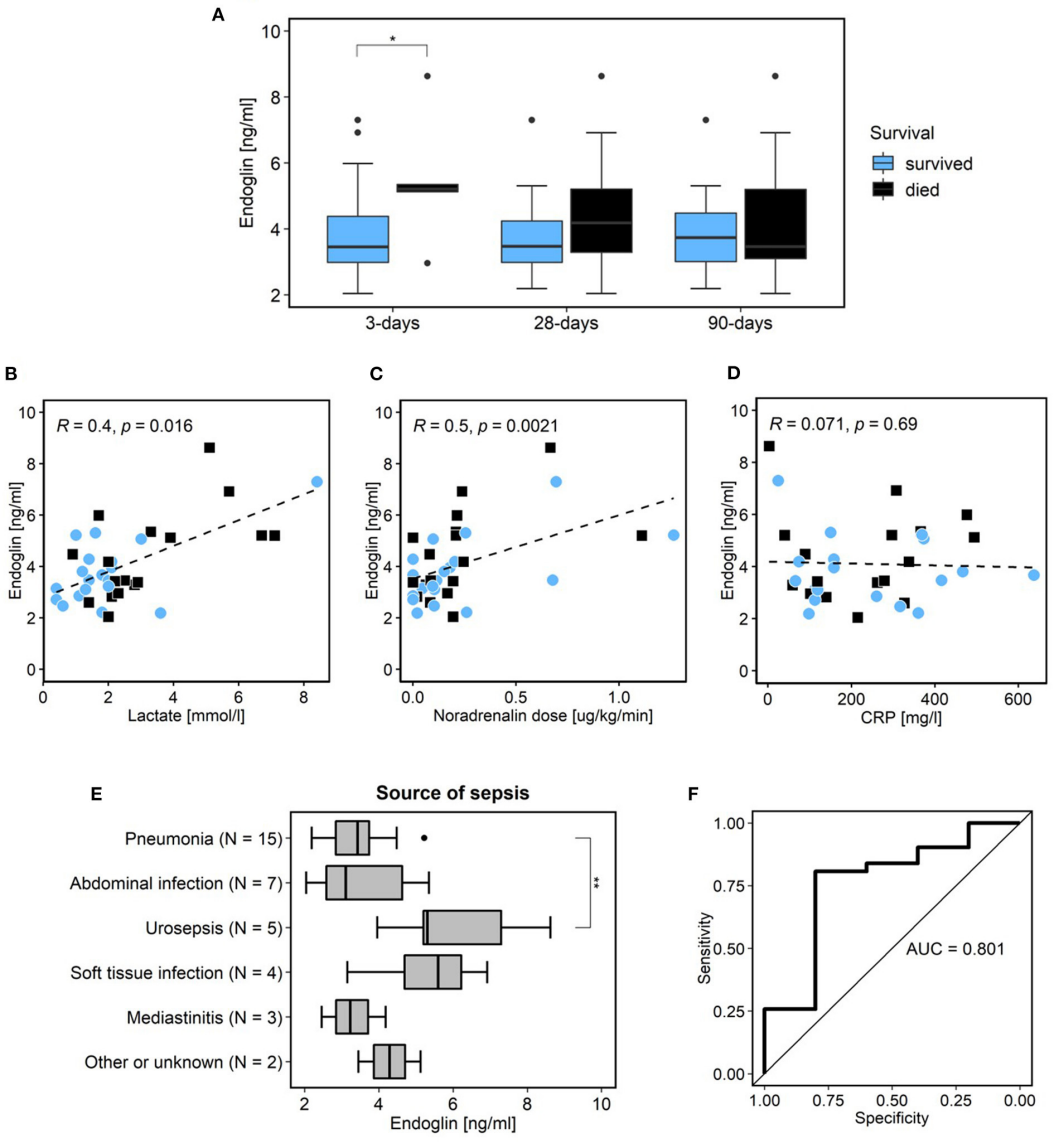
Septic shock cohort. Relationship of patient survival and level of sEng [blue color survivors; black color non-survivors; **(A)**]. Correlations of sEng and lactate **(B)**, noradrenalin dose **(C)**, and CRP [**(D)**; blue dots survivors; black squares non-survivors]. Relation of sEng level and sepsis source **(E)**. Statistical difference between various source of sepsis (Kruskal-Wallis test; *p* = 0.014) and statistical significance between urosepsis and pneumonia (Wilcox test with Holm correction; *p* = 0.007). Receiver operating curve of sEng with calculated AUC for prediction of 3 days mortality **(F)**. *Indicates statistical significance (*p* < 0.05). sEng, soluble endoglin; SOFA, sequential organ failure assessment; CRP, c-reactive protein; AUC, area under the curve. **Indicates statistical significance between urosepsis and pneumonia (Wilcox test with Holm correction; *p* = 0.007).

The sEng level was not statistically significantly elevated in those septic shock patients who died within 28 or 90 days compared to the survivors (*p* = 0.210; *p* = 0.514, respectively; Figure 1A; Table 3); however, the patients with very early mortality (within 3 days of admission) had statistically higher sEng levels than others [5.28 (5.14–7.81) vs. 3.46 ng/mL (2.98–4.38); *p* = 0.019]. The sEng level did not correlate with most clinical data (age, gender, comorbidities, SOFA, creatinine, oxygenation index, CRP etc.; Table 3 and Figure 1D). However, we found a statistically significant correlation between sEng, the lactate level and the required catecholamine dose (*p* = 0.016 and 0.002, respectively; Figures 1B,C; Table 4). The level of sEng also varied according to the source of sepsis (*p* = 0.015); urosepsis and soft tissue infections had a higher median sEng than pneumonia, abdominal and chest infections (Figure 1E). The highest sEng levels were found in patients with urosepsis (8.63 ng/mL in non-survivors and 5.31 ng/mL in survivors).

**Table 3:** Levels of sEng of survived and deceased patients 3, 28, and 90 days after ICU admission.

**Soluble endoglin [ng/mL]**	**Septic shock**			**COVID-19**		
	**Survivors**	**Non-survivors**	***p*-value**	**Survivors**	**Non-survivors**	***p*-value**
3 day mortality	3.46 (2.98–4.38) *n* = 31	5.28 (5.14–7.81) *n* = 6	**0.019**	3.28 (2.71–4.39) *n* = 40	- *n* = 0	-
28 day mortality	3.47 (2.98–4.24) *n* = 19	4.33 (3.31–5.31) n=18	0.210	3.15 (2.50–4.08) n=24	3.54 (3.00–4.49) *n* = 16	0.282
90 day mortality	3.74 (3.01–4.48) *n* = 16	3.47 (3.15–5.21) *n* = 21	0.514	3.13 (2.43–3.94) *n* = 23	3.61 (3.01–4.51) *n* = 17	0.116

Median (1st quartile−3rd quartile) values are presented. For the comparison of surviving and non-surviving patients, p-values of Mann-Whitney test are presented. The bold values indicate statistical significance at *p* < 0.05.

**Table 4:** Correlations and its significance of sEng to other characteristics.

**Spearman's correlation**	**Septic shock**						**COVID-19**					
	**Total**		**Survivors**		**Non-survivors**		**Total**		**Survivors**		**Non-survivors**	
**Characteristic**	**SCC**	***p*-value**	**SCC**	***p*-value**	**SCC**	***p*-value**	**SCC**	***p*-value**	**SCC**	***p*-value**	**SCC**	***p*-value**
Age	0.09	0.597	0.15	0.545	−0.18	0.489	−0.07	0.649	−0.17	0.437	0.09	0.729
SOFA	0.18	0.287	0.15	0.531	0.33	0.200	−0.14	0.378	−0.13	0.559	−0.17	0.517
Noradrenalin dose	0.50	**0.002**	0.44	0.058	0.6	**0.011**	−0.20	0.217	−0.23	0.299	−0.25	0.345
Leukocytes	0.04	0.810	0.06	0.820	−0.16	0.540	0.07	0.656	−0.01	0.949	0.1	0.721
Lactate	0.40	**0.016**	0.31	0.204	0.53	**0.030**	0.13	0.432	0.19	0.383	0.06	0.828
CRP	0.07	0.686	0.03	0.902	0.17	0.521	0.52	**<0.001**	0.6	**0.003**	0.3	0.263
Creatinine	0.21	0.222	0.02	0.928	0.47	0.059	0.04	0.805	−0.01	0.968	0.1	0.700
Bilirubin	0.31	0.068	0.29	0.235	0.4	0.109	−0.18	0.290	−0.14	0.531	−0.44	0.085
Oxygenation index	−0.32	0.060	−0.33	0.176	−0.3	0.239	0.13	0.423	0.28	0.189	−0.07	0.805

Comparison of correlations between Septic and COVID-19 cohorts and between surviving and deceased patients. Correlation strength expressed by coefficient of Spearman's correlation. Relevant statistical significance of correlations presented with p-values. The bold values indicate statistical significance at *p* < 0.05.

SOFA, sequential organ failure assessment; CRP, c-reactive protein; SCC, Spearman's correlation coefficient.

**Figure 2:**
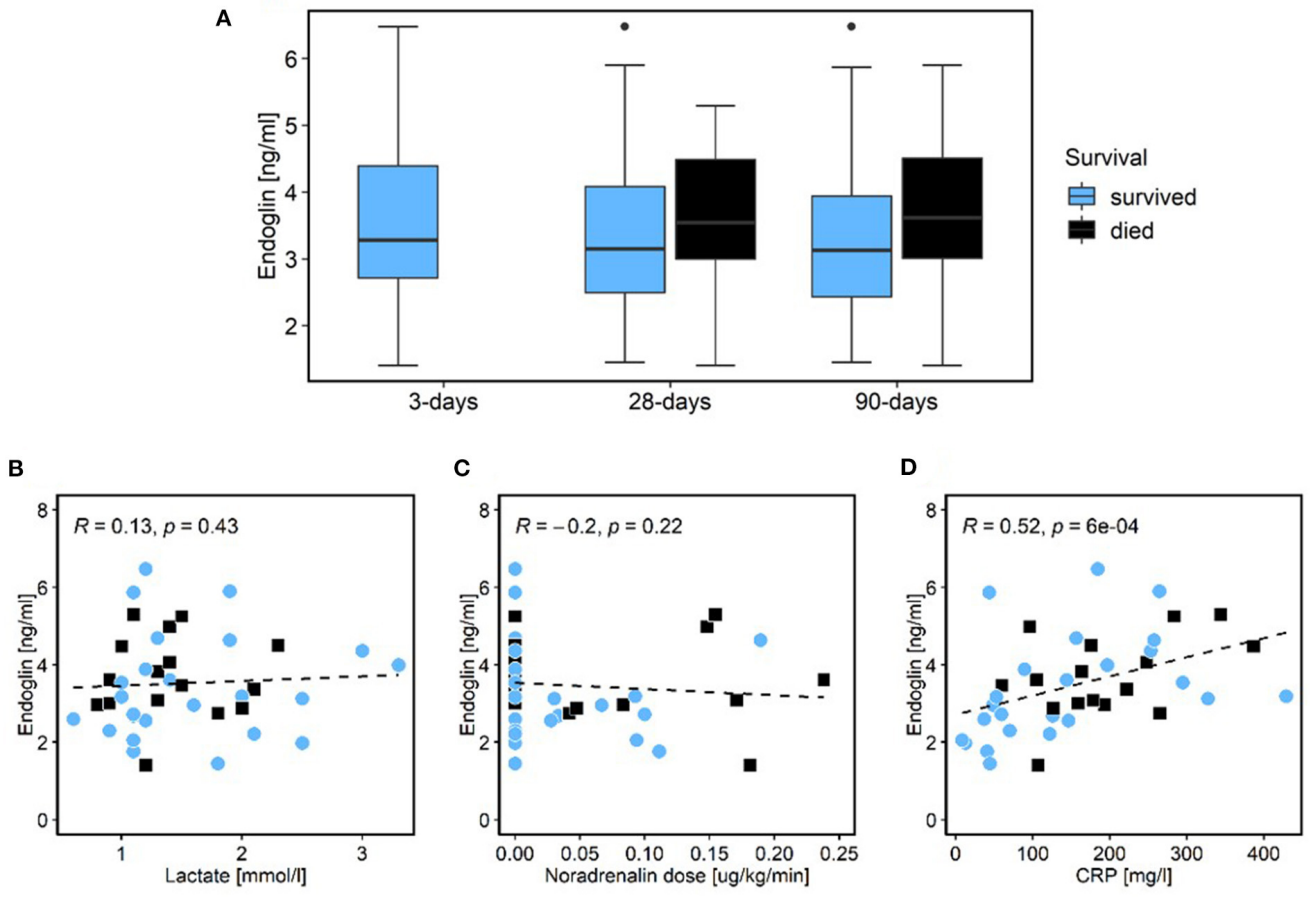
COVID-19 cohort. Relationship of patient survival and level of sEng. None of the patients died within the first 3 days of admission [blue color survivors; black color non-survivors; **(A)**]. Correlations of sEng and lactate **(B)**, noradrenalin dose **(C)**, and CRP [**(D)**; blue dots survivors; black squares non-survivors]. sEng, soluble endoglin; SOFA, sequential organ failure assessment; CRP, c-reactive protein.

In COVID-19 patients, there was no statistically significant difference in sEng levels in relation to D28 and D90 mortality (*p* = 0.282; *p* = 0.116, respectively; Figure 2A; Table 3). The only variable that was statistically correlated with sEng level was C-reactive protein (CRP; *p* = 0.001; Figures 2B–D; Table 4).

## Discussion

The deregulation of endothelial activation in sepsis leads to increased permeability and endothelial barrier dysfunction and is a key mechanism in the development of septic shock. Soluble endoglin, which is released in the increased quantities from endothelial cells during hypoxia and oxidative stress (18) (i.e., factors strongly expressed in septic shock) has been considered by some authors as a marker of the degree of endothelial activation and dysfunction (18, 22–24, 28). Faiotto et al. were the first to describe that sEng is significantly increased in patients experiencing septic shock compared to healthy individuals (29). Moreover, we have recently published that sEng can potentially predict a poor outcome in heterogeneous group of patients with septic shock (25). Here, we report data from a larger and better defined cohort of patients with early onset septic shock. These are patients whose dominant problem is the infection itself and the subsequent reaction to it. Patients from our previous article are not included in this cohort. These were various patients who had often septic shock as a complication of different primary disease (trauma, cardiac arrest, etc.) or have been fighting the infection for a longer time already (25). This is most likely why the results are different. Evaluation with respect to D3, D28, and D90 mortality have proven good prediction of fulminant sepsis (D3 mortality Figure 1F) despite prediction of later mortality.

As noted above, Eng is also expressed in immune cells and has numerous functions in regulating the immune response. It has not been determined whether the increase of sEng in sepsis and septic shock is primarily the result of endothelial activation or a generalized immune response or a different involved factor. Therefore, in the present study, we tested the correlations between sEng and other biomarkers in an attempt to estimate for the first time a link between elevated sEng and other factors [infection (leukocytes, CRP); organ failure (creatinine, bilirubin, the Horowitz index); circulatory failure (catecholamine dose, lactate); severity of condition (SOFA, Lung injury severity score)]. Similarly to sepsis, a severe course of COVID-19 represents a generalized response to infection. However, a shock condition, in the sense of circulatory failure with hypotension and the need for higher doses of catecholamines and severe endothelial dysfunction, is usually not manifested here. Our data show that in the septic cohort, sEng level correlated with the degree of circulatory failure. Therefore, we suggest that in septic shock, sEng primarily reflects the degree of circulatory failure based on endothelial dysfunction and is released mainly from endothelium. Moreover, in patients with a fulminant course of sepsis (D3 mortality), refractory circulatory failure is usually the dominant clinical problem. This is consistent with our finding of significantly higher sEng levels in the early deceased patients. Conversely, in the COVID-19 cohort, sEng did not correlate with mortality, lactate or the catecholamine dose (Figures 2A–C). In septic shock, lactate levels and its change are considered a good prognostic marker (30). In contrast, patients with severe COVID-19 with artificial ventilation do not have high lactate levels and its prognostic value is low (31) which corresponds with our data. We hypothesize that the reason is a lower degree of endothelial dysfunction in COVID-19 patients compared to patients with septic shock. However, elevation of some other markers associated with endothelial function have been already reported in COVID-19 patients with a poor outcome (32). Interestingly, in COVID-19 cohort, sEng levels correlated with CRP levels. For that reason, it is possible that in a situation where endothelial dysfunction is not the main problem, the slight increase in sEng is more likely due to the degree of host-response to infection and or inflammation.

Neither from our data nor from the existing evidence, we are able to say unequivocally that the elevation of sEng accurately reflects the degree of endothelial dysfunction. Undoubtedly, there are multiple mechanisms that can increase sEng levels in such a complex syndrome as sepsis (33). For example, the presence of sepsis-induced cardiomyopathy may also contribute (34).

The fact that sEng is elevated in early deceased septic patients raises the important question whether it is released only as a consequence of the severe condition or it plays some specific role in the increased mortality and could be a potential target for new therapies. To date we are not able to answer this clearly. However, there is evidence that sEng could have a detrimental effect. For example, sEng reduces the activity of the endothelial nitric oxide synthetase (eNOS) and subsequently the natural production of nitric oxide (NO), reduces endothelial turnover, increases the capillary permeability in lungs, kidneys and liver and increases leucocyte adhesion (20, 35). Moreover, not only sEng itself but also the loss of Eng from the endothelium may have its own pathophysiological consequences (33). Regarding the potential pharmacological influence, substances with antioxidant effect such as resveratrol and metformin can reduce the production of sEng (36, 37). In addition, both of these substances have a confirmed protective effect on mortality in sepsis (38, 39).

Changes in sEng levels have been described in association with a variety of comorbidities, in particular, higher comorbidities associated with atherosclerosis, hypertension, diabetes mellitus (DM), obesity, and dyslipidemia (40). In contrast, some studies have described a decrease in obese patients (41). In our data sEng levels did not correlate with age, sex, body mass index (BMI), the number of comorbidities, or the presence of certain chronic disease in either of our two cohorts (Tables 1–3). Moreover, sEng levels were not statistically significantly elevated in patients with any chronic condition associated with persistent endothelial damage and atherosclerosis (DM, ischemic heart disease, peripheral artery disease and ischemic stroke). This means that we did not find any clear bias in our data. Obviously, this may be due to the error associated with the relatively small numbers of patients included in the cohorts.

The dependence of sEng levels on the source of sepsis in the septic cohort is the novel and also interesting result. Patients with a urinary tract infection had the highest sEng levels. The expression of Eng by the urothelium has not been found in any published study to date, but an increased expression by the endothelium has been found in cases of urothelial carcinoma, for example (42). In general, urosepsis has a better survival rate compared to other sources of sepsis (43). In our cohort, the mortality rate of urosepsis was 50%. Therefore, this result is probably not influenced by the severity of sepsis itself.

One limitation of the trial is its monocentric nature and the relatively small numbers of patients included in each cohort. Although the primary objective was not to compare the cohorts with each other, the differences in patients in the septic vs. COVID-19 cohorts pose a limitation too. The COVID-19 patients were younger and had fewer comorbidities compared to the patients with bacterial sepsis. In the COVID-19 patients, severe respiratory failure with a worse oxygenation index was evident (87.5 vs. 143.5); however, the dysfunction of other organs was already expressed to a much lesser extent than in those patients with bacterial sepsis (e.g., creatinine 82 vs. 169 μmol/L; leukocytosis 10.7 vs. 14.6 × 10^9^/L; CRP 151.1 vs. 237.5 mg/L).

In conclusion, our results suggest that soluble endoglin does not predict long-term mortality in patients suffering from septic shock. It is able to predict the fulminant course of sepsis with early (D3) mortality (Figure 1F, AUC 0.801). Its level increases with the severity of circulatory failure or the degree of acute endothelial dysfunction. Therefore, this biomarker could be valuable to identify patients for whom enhanced endothelial dysfunction is a dominant pathophysiological factor in sepsis progression and who would benefit from treatment targeted in this direction (28, 44, 45). However, further studies are necessary to confirm these results and to clarify the role of endoglin in the pathophysiology of sepsis.

## Data availability statement

The raw data supporting the conclusions of this article will be made available by the authors, without undue reservation.

## Ethics statement

The studies involving human participants were reviewed and approved by Local Ethics Committee of the Saint Anne's University Hospital in Brno, Czech Republic. The patients/participants provided their written informed consent to participate in this study.

## Author contributions

VT, MH, and KH: study preparation and design. VT and MH: patient recruitment and sample collection. MS and MŠ: data curation. AM, MHK, and OM: lab analyses. VT and AM: writing—original draft preparation. MH, JF, VŠ, and JP: writing—review and editing. VŠ and JP: supervision. JF: funding acquisition. All authors have read and agreed to the published version of the manuscript.

## Funding

This work has been supported by the Ministry of Health of the Czech Republic (Grant No. NU21-06-00408) and DRO (Institute of Hematology and Blood Transfusion, IHBT, 00023736). JF was supported by the European Social Fund and European Regional Development Fund, Project MAGNET (No. CZ.02.1.01/0.0/0.0/15_003/0000492). The funders had no role in the study design, data collection and analysis, decision to publish, or preparation of the manuscript.

## Conflict of interest

The authors declare that the research was conducted in the absence of any commercial or financial relationships that could be construed as a potential conflict of interest.

## Publisher's note

All claims expressed in this article are solely those of the authors and do not necessarily represent those of their affiliated organizations, or those of the publisher, the editors and the reviewers. Any product that may be evaluated in this article, or claim that may be made by its manufacturer, is not guaranteed or endorsed by the publisher.
